# Exploring the Interactome of Cytochrome P450 2E1 in Human Liver Microsomes with Chemical Crosslinking Mass Spectrometry

**DOI:** 10.3390/biom12020185

**Published:** 2022-01-22

**Authors:** Dmitri R. Davydov, Bikash Dangi, Guihua Yue, Deepak S. Ahire, Bhagwat Prasad, Victor G. Zgoda

**Affiliations:** 1 Department of Chemistry, Washington State University, Pullman, WA 99164, USA; dangibikash@google.com; 2 Department of Pharmaceutical Sciences, Washington State University, Spokane, WA 99202, USA; guihua.yue@wsu.edu (G.Y.); deepak.ahire@wsu.edu (D.S.A.); bhagwat.prasad@wsu.edu (B.P.); 3 Orekhovich Institute of Biomedical Chemistry, Pogodinskaya 10, 119121 Moscow, Russia; victor.zgoda@gmail.com

**Keywords:** chemical crosslinking mass spectrometry (CXMS), alcohol exposure, alcohol-drug interactions, drug metabolism, protein-protein interactions, cytochrome P450, CYP2E1, CYP4A11, CYP4F2, UDP-glucuronosyltransferase

## Abstract

Aiming to elucidate the system-wide effects of the alcohol-induced increase in the content of cytochrome P450 2E1 (CYP2E1) on drug metabolism, we explored the array of its protein-protein interactions (interactome) in human liver microsomes (HLM) with chemical crosslinking mass spectrometry (CXMS). Our strategy employs membrane incorporation of purified CYP2E1 modified with photoreactive crosslinkers benzophenone-4-maleimide and 4-(*N*-succinimidylcarboxy)benzophenone. Exposure of bait-incorporated HLM samples to light was followed by isolating the His-tagged bait protein and its crosslinked aggregates on Ni-NTA agarose. Analyzing the individual bands of SDS-PAGE slabs of thereby isolated protein with the toolset of untargeted proteomics, we detected the crosslinked dimeric and trimeric complexes of CYP2E1 with other drug-metabolizing enzymes. Among the most extensively crosslinked partners of CYP2E1 are the cytochromes P450 2A6, 2C8, 3A4, 4A11, and 4F2, UDP-glucuronosyltransferases (UGTs) 1A and 2B, fatty aldehyde dehydrogenase (ALDH3A2), epoxide hydrolase 1 (EPHX1), disulfide oxidase 1α (ERO1L), and ribophorin II (RPN2). These results demonstrate the exploratory power of the proposed CXMS strategy and corroborate the concept of tight functional integration in the human drug-metabolizing ensemble through protein-protein interactions of the constituting enzymes.

## 1. Introduction

The primary role in the metabolism of drugs and other xenobiotics in the human body is played by the cytochrome P450 ensemble, which is responsible for the metabolism of over 75% of all marketed drugs and new drug candidates [1,2]. The functional versatility of the P450 ensemble is achieved through the presence of over a dozen P450 species differing in their substrate specificity. Although the premise that the properties of this ensemble represent a simple aggregate of those of the constituting P450 enzymes continues to be the cornerstone of analyzing the routes of drug metabolism, its validity is essentially compromised [3,4,5,6,7]. A significant complexity in the relationship between the composition of the pool of P450s in the human liver and the system-wide properties of the drug-metabolizing system is brought in by the consequences of protein-protein interactions of its constituents, including the formation of heteromeric complexes of multiple P450 species and the interactions of P450s with other drug-metabolizing enzymes and regulatory proteins [8,9,10,11,12].

Of particular significance is the role of protein-protein interactions of P450s in altering human drug metabolism by chronic alcohol exposure. The multifold increase in P450 2E1 (CYP2E1) content in the liver and other tissues observed in both alcoholics and moderate alcohol consumers represents one of the most important effects of alcohol on protein expression [13,14]. The importance of this enzyme for the mechanisms of hepatotoxicity is well recognized [13]. Conversely, the involvement of CYP2E1 in the instances of alcohol-drug interactions is commonly considered insignificant due to its minor role in drug metabolism [15,16].

However, the impacts of the alcohol-induced increase in CYP2E1 content on drug metabolism and other functions of the P450s appear to be underestimated. The effects of interactions of CYP2E1 with other P450s provide the most likely explanation for the alcohol-induced increase in the metabolism of diazepam and doxycycline [17,18,19], the substrates of CYP3A, or phenytoin, tolbutamide, and warfarin [20,21] metabolized primarily by CYP2C9. Conclusive evidence of a direct cause-to-effect relationship between alcohol-dependent induction of CYP2E1 and the effects of this kind is provided by our studies of the impact of CYP2E1 on the activity of CYP3A4, CYP1A2, and CYP2C19 [22,23].

The present study explores the network of protein-protein interactions (the interactome) of CYP2E1 in the endoplasmic reticulum (ER) of liver cells with a novel strategy of chemical crosslinking mass-spectrometry (CXMS) that employs membrane incorporation of the bait protein (CYP2E1) modified with photoreactive crosslinkers benzophenone-4-maleimide (BPM) and 4-(*N*-succinimidylcarboxy)benzophenone (BPS). Interaction of BPM with proteins results in attaching benzophenone moiety to thiol groups of surface-exposed cysteine residues, while BPS reacts with ε-amino groups of lysines, as well as with the N-terminal α-amino group. Exposure of the protein-attached benzophenone moiety to near-UV (330–365 nm) light results in n-π* electronic transition. It breaks the C=O double bond and generates a benzophenone diradical, which triplet state attacks the adjacent C-H bonds and forms unspecific covalent crosslinks with the nearby proteins [24].

**Figure 1:**
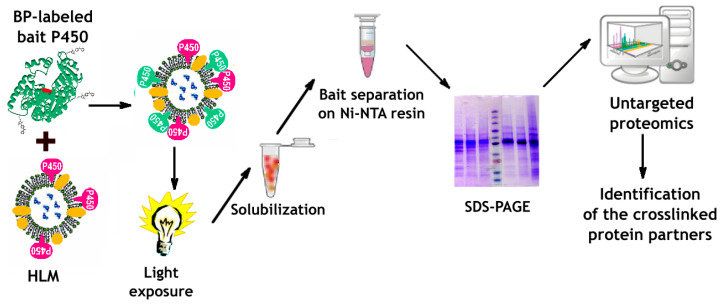
The workflow of the proposed CXMS strategy.

The workflow of our strategy is illustrated in Figure 1. It involves the incorporation of BPM- or BPS-activated bait protein (CYP2E1) into microsomal membranes followed by light exposure of bait-incorporated samples. After solubilizing the microsomal membrane with detergent, the His-tagged bait and its crosslinked aggregates are isolated using Ni-NTA agarose and subjected to SDS-PAGE. Analyzing the individual zones of the gel slabs with the toolset of untargeted proteomics allows detecting crosslinked complexes of the bait with other microsomal proteins and thus exploring the network of its protein-protein interactions.

This new approach allowed us to detect the crosslinked complexes of CYP2E1 with such drug-metabolizing enzymes (DMEs) as the P450s 2A6, 2C8, 3A4, 4A11, and 4F2, and UDP-glucuronosyltransferases (UGTs) 1A and 2B. These results demonstrate the high exploratory power of the proposed strategy and corroborate the concept of tight functional integration in the human drug-metabolizing ensemble through protein-protein interactions of the constituting enzymes.

## 2. Materials and Methods

### 2.1. Chemicals

BPM and Igepal CO-630 were the products of Sigma Aldrich Inc (St. Louis, MO, USA). BPS was obtained from Chem-Impex Intl. Inc. (Wood Dale, IL, USA). 7-Ethoxy-4-trifluoromethylcoumarin and 7-Hydroxy-4-trifluoromethylcoumarin were the products of Molecular Probes Inc. (Eugene, OR, USA), now a part of Thermo Fisher Scientific (Waltham, MA, USA). All other reagents were of ACS grade and used without additional purification.

### 2.2. Protein Expression and Purification

C-terminally His-tagged and N-terminally truncated Δ3–20 CYP2E1 [25] was expressed in *E. coli* TOPP3 cells and purified as described earlier [23].

### 2.3. Pooled Human Liver Microsomes

In this study, we used two different lots of Human Liver Microsomes (HLM) obtained from 50 donors (mixed gender), namely the lots LFJ and LBA designated hereafter as HLM (LBA) and HLM (LFJ). These preparations were purchased from BioIVT (Westbury, NY, USA). The relative abundances of 11 major P450 species in both lots were characterized in our earlier study [26]. The supplier-provided characterization of both lots may be found in the Supplementary Materials to the above publication [26]. 

### 2.4. Characterization of the Content of Protein and Cytochromes P450 in HLM

The protein concentrations were determined with the bicinchoninic acid procedure [27]. The total concentration of P450s in HLM was determined with a variant of the “oxidized CO versus reduced CO difference spectrum” method described earlier [23]. The concentration of CPR in microsomal membranes was determined based on the rate of NADPH-dependent reduction of cytochrome c at 25 °C, and the effective molar concentration of CPR was estimated using the turnover number of 3750 min^−1^ [23].

### 2.5. Modification of CYP2E1 with BPM and BPS

The reaction with BPM was performed in 0.5 M K-phosphate buffer, pH 7.4, containing 20% glycerol. The modification with BPS was carried out in 0.125 M K-phosphate buffer, pH 8.2, containing 10% glycerol. Buffer replacement was achieved by a passage through a spin-out column of Bio-Gel P6 (Bio-Rad, Hercules, CA, USA) and followed with a dilution to the final protein concentration of 10 µM. The resulting protein solution was placed into a conic glass vial and saturated with argon through gentle bubbling of the gas. In the case of BPS, at this stage, we supplemented the incubation mixture with 0.2% Igepal CO-630 added as 10% solution in the same buffer. The modifying reagent (BPM or BPS) was added to the desired molar ratio to the protein (see Section 3.1) as a 10 mM solution in dimethylformamide. The incubation vial was flushed with argon, tightly closed, and set for overnight incubation in the dark at 4 °C with continuous stirring. In the case of BPM, the reaction was stopped by adding reduced glutathione to the concentration of 1 mM. The detergent present in the incubation mixture with BPS was removed using a DetergentOUT^TM^ spin column (G-Biosciences, St Louis, MO, USA). The protein was concentrated to 25–30 µM with the use of a Centrisart I MWCO 100 kDa concentrator (Sartorius AG, Göttingen, Germany) and passed through a spin-out column of Bio-Gel P-6 equilibrated with the protein storage buffer (0.1 M Hepes-HCl, 10% glycerol, 150 mM KCl).

### 2.6. Incorporation of CYP2E1 into HLM

Incorporation of benzophenone modified CYP2E1 into HLM was performed following the procedure described previously [23,26,28]. Undiluted suspensions of HLM (20–25 mg/mL protein, 10–13 mM phospholipid) in 125 mM K-Phosphate buffer containing 0.25 M sucrose (sucrose buffer) with benzophenone-modified protein or intact purified CYP2E1 (in control experiments) for 16–20 h in the dark at 4 °C under an argon atmosphere at continuous stirring. The incubation time was adjusted based on monitoring the incorporation process with FRET-based techniques [23,26,28]. The protein being incorporated was added in the amount of one molar equivalent to the endogenous P450 present in HLM. Following the incubation, the suspension was diluted 4–8 times with the sucrose buffer and centrifuged at 150,000× *g* for 90 min at 4 °C. Finally, the pellet was resuspended in the same buffer to the protein concentration of 15–20 mg/mL.

### 2.7. Probing the Catalytic Activity of BPM- and BPS-Modified CYP2E1

To obtain catalytically active reconstituted systems with CYP2E-BPM and CYP2E1-BPS, we incorporated these proteins into insect cell microsomes (Supersomes^TM^, Corning Inc., Corning, NY, USA) containing cDNA-expressed human NADPH-cytochrome P450 reductase (CPR) along with human cytochrome b_5_ (SS(CPR+b5)). As reference points, we used SS(CPR+b5) with incorporated unmodified CYP2E1 (SS(CPR+b5)2E1) and commercial Supersomes containing cDNA-expressed full-length CYP2E1 (SS(2E1)). The preparations of SS(CPR+b5) and SS(2E1) (product numbers 456244 and 456206, respectively) were the products of BD Gentest, now a part of Corning Life Sciences (Tewksbury, MA, USA).

Incorporation of purified CYP2E1 and its BPM and BPS derivatives into SS(CPR+b5) was performed with a procedure similar to that described above for CYP2E1 incorporation into HLM. The amount of the proteins added to SS(CPR+b5) was adjusted to achieve the 2:1 molar ratio to the content of CPR calculated based on the rate of cytochrome c reduction (see above).

The rate of oxidative deethylation of 7-Ethoxy-4-trifluoromethylcoumarin (7-EFC) was determined with a real-time fluorescence assay [28] using Cary Eclipse fluorescence spectrophotometer equipped with a thermostated cell holder and magnetic stirrer. The assays were performed in 0.1 M Na-Hepes buffer, pH 7.4, under continuous stirring at 30 °C in 5 × 5 mm quartz cells at the concentration of Supersomes of 3–12 nM (per CPR content). The concentration of 7-EFC varied from 1 to 130 µM. The reaction was started by adding a 20 mM solution of NADPH to the concentration of 100 µM. The process of formation of 7-Hydroxy-4-trifluoromethylcoumarin (7-HFC), the fluorescent product of the reaction, was followed by real-time monitoring of the increase in fluorescence at 495 nm (20 nm slit width) with excitation at 405 nm (20 nm slit width). The rate of product formation was determined from the linear approximations of the initial 120–180 s portions of the kinetic curves using the calibration coefficient determined from a series of fluorescence intensity measurements at stepwise additions of 2 mM solution of 7-HFC to the reaction mixture containing no NADPH and 7-EFC. 

### 2.8. Crosslinking of the Bait Protein and Its Subsequent Isolation from HLM

The suspension of HLM with incorporated benzophenone-modified CYP2E1 was diluted to the protein concentration of 5 mg/mL by argon-saturated sucrose buffer and placed into a 1 × 1 cm optical quartz cell. The cell was flushed with argon gas, tightly closed, and exposed to a broadband UV-Vis light using a 6427 Xe flash lamp light source (Oriel Instruments, Stratford, CT, USA) operating at 75 Hz flash rate and maximal power. After 2 h of light exposure, the suspension was centrifuged at 105,000× *g* for 90 min. The pellet was resuspended in 1 mL of 0.125 M K-phosphate buffer, pH 7.4, containing 20% glycerol and 0.5% Igepal CO-630. The mixture was incubated for 2 h at 4 °C at continuous stirring and centrifuged at 105,000× *g* for 90 min.

The supernatant was applied to a 0.2 mL HisPur™ Ni-NTA Spin Column (Thermo Fisher Scientific, Waltham, MA, USA) equilibrated with the same buffer. Following one hour of incubation of the closed column under periodical shaking, the column was washed with multiple subsequent 1 mL portions of the same buffer containing 0.5% CHAPS until the optical density of the flow-through at 280 nm decreases below 0.025 OD units. The bound protein was eluted with 500 mM K-Phosphate buffer, pH 7.4, containing 20% glycerol, 0.5% CHAPS, and 250 mM imidazole. The detergent was removed using a Bio-Beads SM-2 resin (Bio-Rad, Hercules, CA, USA). The protein solution was concentrated to 10–20 mg/mL using a Centrisart I MWCO 100 kDa concentrator (Sartorius AG, Göttingen, Germany).

The control experiments were performed with unmodified CYP2E1 subjected to incorporation in the same microsomal preparations and subsequent isolation following the procedure described for the benzophenone-activated protein.

### 2.9. Untargeted Proteomics Assays

The proteins extracted from Ni-NTA resin were subjected to SDS-PAGE on 4–15% Mini-PROTEAN^®^ TGX™ Precast Protein Gels (Bio-Rad, Hercules, CA, USA). The Broad Multi Color Pre-Stained Protein Standard from GenScript (Piscataway, NJ, USA) was used for calibration. The gels were stained with Coomassie Brilliant Blue R-250 Staining Solution (Bio-Rad) and subjected to fragmentation, as described under Results. The resulting gel fragments were subjected to untargeted proteomic analysis. 

The HLM(LFJ) samples were analyzed in Viktor Zgoda’s laboratory. The fragments of the SDS-PAGE slabs were first washed twice with 10% acetic acid and 20% ethanol for 10 min, and then five times with HPLC grade water for 2 min and two times with 40% acetonitrile and 50 mM NH4HCO3. After drying with acetonitrile and on-air, the gel fragments were digested by trypsin using a previously described protocol [29]. MS analysis was performed with a Q Exactive HF mass spectrometer (Q Exactive HF Hybrid Quadrupole-Orbitrap™ Mass spectrometer, Thermo Fisher Scientific, Waltham, MA, USA) as described earlier [29].

The HLM(LBA) samples were analyzed in the laboratory of Bhagwat Prasad. Pre-digestion treatment of the gel fragments was performed following the procedure described in [30]. LC-MS/MS analysis was performed with nanoLC coupled to a Q Exactive HF hybrid Quadrupole-Orbitrap™ mass spectrometer (Thermo Fisher Scientific, Waltham, MA, USA) [31].

In both cases, the obtained raw data were processed using the MaxQuant software (version 2.0.1.0 [32]) with the built-in search engine Andromeda. Protein identification was performed against the complete human proteome provided by Uniprot. Carbamidomethylation of cysteines was set as fixed modification, and protein N-terminal acetylation, as well as oxidation of methionines, was selected as a variable modification for the peptide search. The false discovery rates (FDR) for protein identifications were set to 1%.

## 3. Results

### 3.1. Modification of CYP2E1 with Benzophenone Derivatives 

Incubation of CYP2E1 with BPM in the conditions described under the Materials and Methods section resulted in the incorporation of up to three molecules of label per molecule of the enzyme. No increase in the degree of modification or precipitation of denatured protein was observed at increasing the amount of added BPM up to six molar equivalents to the protein. Therefore, there are only three cysteine residues per molecule of CYP2E1 that BPM can modify without protein unfolding or inactivation. The BPM-crosslinking experiments described below were performed with the protein labeled at the molar ratio of 2.4–2.7.

Successful modification of CYP2E1 by BPS required the presence of detergent (Igepal CO-630, 0.2%). Under these conditions, incubation of CYP2E1 with nine molar equivalents of BPS did not cause any protein precipitation. It resulted in the incorporation of seven molecules of the probe per molecule of the protein.

**Figure 2:**
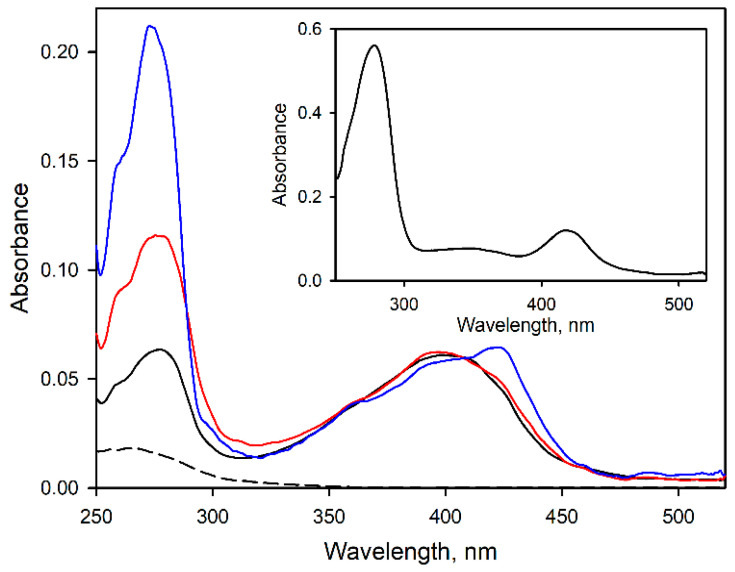
Modification of CYP2E1 with BPM and BPS. The graph shows the absorbance spectra of purified CYP2E1 (solid black line) and CYP2E1 modified with BPM (red) and BPS (blue). The black dashed line shows the spectrum of absorbance of 1 µM BPM. The inset shows the spectrum of the BPM-modified protein eluted from the Ni-NTA resin after the crosslinking experiment. The spectrum was taken in the presence of 0.25 M imidazole. All spectra were normalized to correspond to the heme protein concentration of 1 µM.

Figure 2 exemplifies the spectra of UV-Vis absorbance of the purified CYP2E1 and its adducts with BPM and BPS. While modification with BPM at 3:1 molar ratio did not result in any noticeable displacement of the spin equilibrium of the heme protein or its conversion into the inactivated P420 state, incorporation of 7 molecules of BPS per CYP2E1 molecule resulted in a moderate (up to 25%), formation of the P420 form of the heme protein. Similar to what was observed with CYP2E1-BPM adducts, the spin state of the heme protein (~70% of the high-spin state) remained unaffected upon its modification with BPS. 

### 3.2. Effect of Modifications of CYP2E1 by BPM and BPS on the Functional Properties of the Enzyme

To probe the effect of CYP2E1 modification by benzophenone derivatives on the functional properties of the enzyme, we incorporated CYP2E1-BPM and CYP2E1-BPS into insect cell microsomes (Supersomes^TM^) containing cDNA-expressed human NADPH-cytochrome P450 reductase (CPR) along with human cytochrome b_5_ (SS(CPR+b5)) and studied their activity in deethylation of 7-Ethoxy-4-trifluoromethylcoumarin (7-EFC). Although this fluorogenic substrate is commonly used as a CYP2B6-specific probe, it can also be metabolized by CYP2E1 with a reasonable turnover rate [25,33,34]. To obtain the appropriate reference points, we also studied 7-EFC metabolism by SS(CPR+b5) with incorporated unmodified CYP2E1 (SS(CPR+b5)2E1) and commercial preparation of Supersomes containing cDNA-expressed full-length CYP2E1 (SS(2E1)). To compare the turnover rates in these preparations, we normalized the datasets on the concentration of CPR, which is the limiting component in CYP2E1-incorporated SS(CPR+b5) samples. The only exception is SS(2E1), where the concentrations of CYP2E1 and CPR in SS(2E1) were equal to 2 and 3.6 µM, respectively. Since the reductase was present in excess in this system, its turnover rate was normalized per the heme protein content.

**Figure 3:**
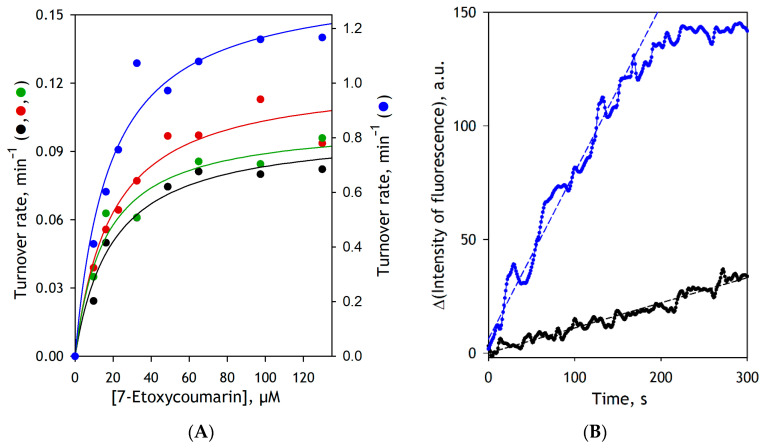
The effect of modification of CYP2E1 with BPM and BPS on the kinetics of deethylation of 7-EFC by the enzyme incorporated into Supersomes. (**A**) The substrate-saturation profiles obtained with SS(2E1) (black), SS(CPR+b5)2E1 (red), SS(CPR+b5)2E1-BPM (green) and SS(CPR+b5)2E1-BPS (blue). The shown datasets represent averages of two (SS(2E1), SS(CPR+b5)2E1, and SS(CPR+b5)2E1-BPM) or four (SS(CPR+b5)2E1-BPS) individual experiments. The turnover rates are calculated per the CPR content, except for the SS(2E1) dataset, which is normalized on the concentration of CYP2E1. Solid lines show the approximation of the experimental datasets with the Michaelis-Menten equation. (**B**) Kinetic traces obtained with SS(2E1) (black) and SS(CPR+b5)2E1BPS (blue) at 7-EFC concentration of 130 µM. The concentration of microsomal CPR in the incubation mixture was equal to 0.06 and 0.01 µM, respectively. Dashed lines show linear approximations of the initial portions (first 180 s) of the traces.

The parameters of 7-EFC metabolism by these microsomal preparations are summarized in Table 1, and the respective substrate saturation profiles along with the representative kinetic curves are exemplified in Figure 3. As seen from these data, similar to that was shown earlier with Supersomes containing rat CPR [35], incorporation of purified CYP2E1 into SS(CPR+b5) allows to obtain a catalytically active reconstituted system. Its kinetic parameters in the reaction of 7-EFC deethylation do not exhibit any substantial difference from those characteristics of the Supersomes containing the baculovirus-expressed full-length enzyme (Table 1). Notably, the substitution of unmodified CYP2E1 with CYP2E1-BPM has a negligible effect on the *K*_M_ and *V*_max_ values. As seen from Table 1 and Figure 3A, the BPM-modified enzyme is as active as unmodified CYP2E1, and its affinity to the substrate remains unaffected.

The most surprising outcome of these experiments is an observation of a multifold activation of CYP2E1 by its modification with BPS (Table 1, Figure 3A). Notably, in contrast to unmodified CYP2E1, where the rate of 7-EFC deethylation remains constant for at least 10 min, the BPS-modified enzyme becomes inactivated in 3–4 min after the addition of NADPH (Figure 3B). As the attachment of BPS modifies the protein amino groups, it is very unlikely that the mechanism of this activation involves the stabilization of CYP2E1 complexes with CPR. The P450-CPR interactions are known to have an electrostatic nature and involve surface-exposed amino groups of the heme protein [36]. The observed activating effect of BPS modification instead suggests that the modification of the CYP2E1 amino groups by BPS may affect the active site architecture or modify the enzyme’s conformational dynamics during the catalytic cycle. These changes boost the catalytic activity at the expense of decreased catalytic stability. The increase in the rate of 7-EFC deethylation by CYP2E1 caused by modifying its amino groups by BPS does not seem too unprecedented given its low catalytic efficiency in this reaction. For instance, a similar multifold activation of 7-EFC metabolism by CYP2E1 was caused by an F477V substitution in the enzyme’s active site [25].

Taken together, the above observations demonstrate that the modifications of CYP2E1 with BPM and BPS do not inactivate the enzyme and do not prevent its incorporation into the microsomal membrane or interfere with its interactions with CPR.

### 3.3. Incorporation of Modified CYP2E1 into HLM, Its Photo-Activated Crosslinking, and Subsequent Isolation from the Membranes

Similar to that shown earlier for unmodified CYP2E1 [26], incubation of BPM- and BPS-labeled protein with HLM at 1:1 molar ratio of the added CYP2E1 to endogenous microsomal P450 content resulted in the incorporation of 70–80% of the added protein. This estimate is based on determining the CYP2E1 content in the supernatant fraction after separating CYP2E1-enriched microsomes by differential centrifugation.

**Figure 4:**
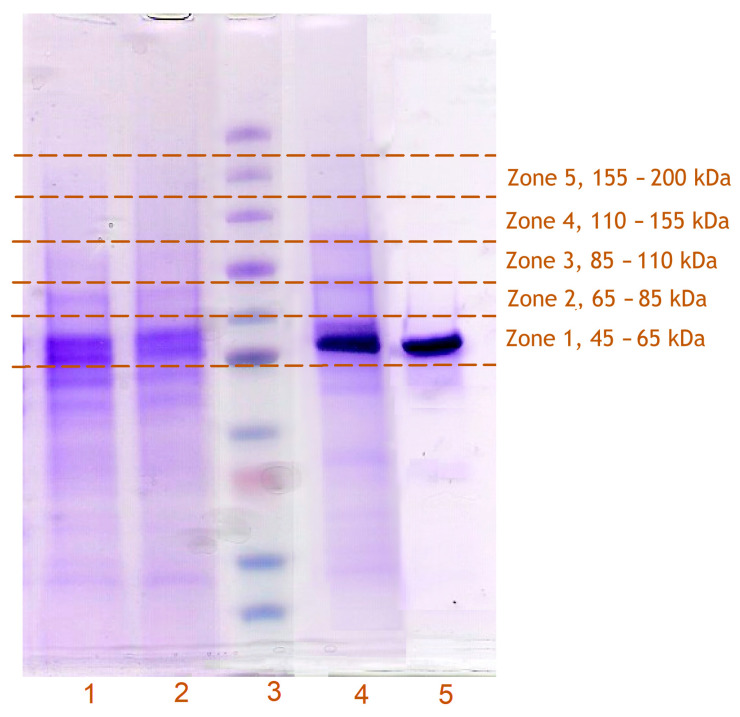
The scheme of fragmentation of SDS-PAGE slabs for MS/MS analysis. The SDS-PAGE slab shown in the figure exemplifies the lanes loaded with proteins obtained in crosslinking experiments with HLM(LFJ) using BPM-CYP2E1 (1) and BPS-CYP2E1 (2) as a bait protein and a control experiment with the same microsomes subjected to incorporation and isolation of unlabeled CYP2E1 (4). Lanes 3 and 5 correspond to the calibrating protein ladder (5, 15, 30, 35, 50, 65, 95, 130, 175, and 270 kDa) and the purified CYP2E1 protein, respectively.

After the light exposure of the bait-containing microsomes and solubilization of the membranes with detergent (Igepal CO-630, 0.5%), the extracted CYP2E1 binds quantitatively to Ni-NTA resin. Washing the resin with 30–35 column volumes of the CHAPS-containing buffer (see Section 2) decreased the protein absorbance band in the flow-through from the initial 1.2 to approximately 0.025 OD units. Eluting the bound protein with 0.25 M imidazole allowed recovering labeled CYP2E1 in the amount of up to 50% of that taken for the experiment. The UV/Vis absorbance spectrum of the eluate (Figure 2, inset) indicated the presence of considerable amounts of crosslinked or non-specifically bound proteins. Consequently, SDS-PAGE assays revealed several noticeable bands corresponding to the proteins with molecular masses different from CYP2E1 (Figure 4). As seen from Figure 4, the pattern of the bands observed in the control experiment with unlabeled CYP2E1 reveals no noticeable difference from that seen with the benzophenone-activated CYP2E1 suggesting that both samples may contain non-specifically bound proteins.

### 3.4. Identification of CYP2E1-Crosslinked Proteins with Untargeted Proteomics

To identify potential crosslinks of the benzophenone-activated CYP2E1 with other proteins, the fragments of the SDS-PAGE slabs corresponding to the molecular masses equal or higher than that of CYP2E1 (57 kDa) were subjected to untargeted proteomics assays. The scheme of fragmentation of the SDS-PAGE slabs for this analysis is illustrated in Figure 4.

To ensure reliable identification of crosslinked proteins and minimize the likelihood of false positives, we compared the results of six individual CXMS assays, which included three separate CXMS assays with each of the BPM and BPS crosslinkers. Two assays were performed with HLM (LFJ) and one with HLM (LBA).

The proteomics analysis of the SDS-PAGE fragments revealed the presence of multiple microsomal and cytoplasmic proteins. Their complete list, along with the values of peak intensities observed in the individual gel zones in each of the six CXMS experiments, may be found in Table S1 in the Supplementary Materials. In our analysis, we normalized the peak intensities by dividing them by the total intensity for all proteins found in each zone. Thus, the values shown in Table S1 (Supplementary Materials) are expressed as the percent contribution of each protein to the total.

Most of the found peptides correspond to the proteins located in the microsomal membrane or the microsomal lumen. Among those proteins, the most abundant were CYP2E1, CES1 (liver carboxylesterase 1), and P4HB (protein disulfide isomerase). According to the peptide peak intensity, these three proteins contribute to over 60% of all proteins found.

The identification of the potentially crosslinked proteins was based on the analysis of their distribution between the different zones of the SDS-PAGE lanes. Theoretically, all proteins present in the gel lane must be found in the zones corresponding to their molecular masses at no crosslinking. All P450s, UGTs, and most of the other microsomal membranous proteins of interest (NADPH-cytochrome P450 reductase, heme oxygenase 1, microsomal epoxide hydrolase, flavin-containing monooxygenases, etc.) have molecular masses between 45 and 85 kDa. They must be therefore found in zones 1 and 2. Their appearance in the higher-molecular-weight zones indicates crosslinking with the bait protein.

Analyzing Table S1 (Supplementary Materials), one can see that many abundant microsomal proteins appear in the gel zones corresponding to the molecular masses higher than their own, even in the control samples. These occurrences are caused by the low resolving power of the SDS-PAGE combined with the high sensitivity of the LC-MS/MS technique. Due to these circumstances, the only possible way to judge the presence of crosslinked proteins in SDS-PAGE lanes is by comparing the normalized peak intensities observed in the crosslinked samples with those found in the control experiments without crosslinking.

In our preliminary screening of the CXMS results, we analyzed the ratios of the normalized peak intensities observed in zones 3 and 4 (molecular masses of 85–155 kDa) to those detected in zones 1 and 2 (45–85 kDa). The ratios calculated for the crosslinked samples were compared with those obtained with the control samples where non-activated CYP2E1 was subjected to the same procedure as in the experiments with BPM- and BPS-labeled CYP2E1. The instances where the ratio observed in the crosslinked sample was higher than that in the respective control were considered to indicate crosslinking.

We calculated these ratios for all microsomal membranous proteins with molecular masses of 45–85 kDa found in the samples. We picked over the proteins where these instances were encountered in at least four out of six individual CXMS experiments. The resulting list of potential crosslinking partners of CYP2E1 is given in Table 2. Besides several P450 species and a set of UGTs, this list includes such microsomal membranous proteins as fatty aldehyde dehydrogenase (FALDH, gene name ALDH3A2), epoxide hydrolase 1 (EPHX1), disulfide oxidase 1α (Ero1α oxidase, ERO1L), flavin-containing monooxygenase FMO3, and ribophorin II (RPN2), a part of *N*-oligosaccharyltransferase complex. Three of these five proteins (FALDH, EPHX1, and FMO3) are immediately involved in or closely related to xenobiotic metabolism in the liver.

Our further analysis involved a closer examination of the patterns of protein distribution between the SDS-PAGE zones. Besides the set of the proteins found in the first round of screening (Table 2), we also analyzed the peptides corresponding to cytochrome b_5_ (CYB5A) and progesterone receptor membrane component 1 (PGRMC1). These potential P450 interaction partners have molecular masses below 45 kDa and were not therefore considered in the first round of screening. To avoid possible oversight of CYP2E1-interacting P450 and UGT species, we complemented the list of proteins under analysis with all species of these DMEs found in the crosslinked samples. In addition, we also analyzed the distribution of liver carboxylesterase 1 (CES1) and protein disulfide isomerase (P4HB) between the gel zones. These two highly abundant proteins were used as no-crosslinking references, as they are located in the ER lumen and are therefore unable to interact with P450s unless the ER membrane is destroyed.

In this analysis, we normalized the relative summarized peak intensities of all peptides corresponding to each protein to the total of their intensities observed in all five analyzed gel zones. The resulting values characterize the distribution of each particular protein between the zones. At the final analysis step, we calculated the ratio of these double-normalized values observed in crosslinking experiments to those obtained in the control experiments with unlabeled CYP2E1.

**Figure 5:**
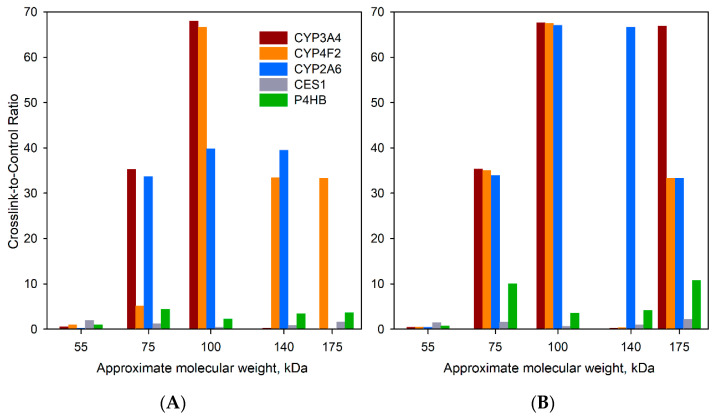
Distribution of potentially crosslinked proteins between the individual bands of the SDS-PAGE lanes obtained in the experiments with BPM-CYP2E1 (**A**) and BPS-CYP2E1 (**B**). The data shown in the graph represent the averages of three individual experiments with HLM(LFJ) and HLM(LBA) summarized in Table 3. The Y-axis of the plot corresponds to the ratio of normalized apparent protein abundance in the crosslinked sample to that observed in control. The ordinate of the graph shows the approximate averaged molecular weights of all proteins found in each band.

The examples of the profiles of crosslinking-to-control ratio calculated in this way are shown in Figure 5. These profiles represent the averages of three individual profiles calculated for BPM- (panel A) and BPS-intermediated (panel B) crosslinking. To calculate these averages, we arbitrarily assigned the value of 100 to the instances where no protein was found in the control while being present in the crosslinked sample. The contrasting difference of the profiles obtained for CYP3A4, CYP4F2, and CYP2A6 from those calculated for CES1 and P4HB suggests an abundant formation of crosslinked aggregates of the former three proteins with the bait (CYP2E1). As seen from Figure 5, the averaged crosslinking-to-control ratios for CYP3A4, CYP2F2, and CYP2A6 in the SDS-PAGE zones corresponding to molecular masses >65 kDa are up to two orders of magnitude higher than those observed with CES1 and P4HB, non-crosslinking reference proteins. Notably, the results obtained with two different crosslinkers—BPM and BPS exhibit similar patterns. In both cases, the profiles obtained with P450 enzymes display a pronounced maximum in the zone corresponding to the molecular masses of 85–110 kDa, indicating a predominant formation of dimeric crosslinks. Interestingly, the profiles obtained with BPS suggest a more extensive formation of trimeric aggregates. This observation is consistent with the higher degree of labeling in BPS-modified CYP2E1 compared to its BPM-modified counterpart (seven benzophenone groups per protein molecule with BPS, as compared to three in the case of BPM).

Results of calculating the crosslinking-to-control ratio for the proteins picked over at the first step of screening (Table 2) along with CYB5A, PGRMC1, and all detected P450 and UGT species in all six individual experiments (three experiments with each of the two crosslinking agents) are summarized in Table 3. Analyzing the data presented in this table, the reader may see that, despite general conformity between the results of the individual CXMS assays, there are some noticeable variations in the distribution of some proteins (e.g., CYP4A11) between the gel zones. Besides the differences of the two HLM preparations in protein composition and dissimilarities in the crosslinking patterns of BPM and BPS, these differences may be caused by variations in the protein loads in the LC-MS/MS assays (see row 750 in Table S1, Supplementary Materials), the limited resolution of SDS-PAGE technique and unavoidable slight variations in the patterns of fragmentation of the gel slabs. Importantly, in most cases where the abundance of proteins in the zones with molecular masses higher than their own were higher in the crosslinked samples than in the controls, these proteins were utterly missing in the respective control zones. These instances are identified in Table 3 with infinity sign (∞).

In further analysis, we identified the proteins that exhibited the crosslinking-to-control ratio higher than 30 in any of the gel zones corresponding to molecular masses higher than that of the respective protein itself (zones 2–5 for P450s and UGTs, zones 3–5 for NCPR and zones 1–5 for cytochrome b_5_ and PGRMC1). The threshold of 30 was chosen based on the highest value encountered with no-crosslinking reference proteins (the value of 28 observed with P4HB in Zone 2 of the HLM(LBA) sample). The proteins matching this criterion in four or more individual experiments were considered the most probable protein–protein interaction partners of CYP2E1. Table 3 does not show the results for most proteins exhibiting less than three hits over the six experiments. The only exception is NADPH-cytochrome P450 reductase. The lack of steadily detected crosslinks of this electron-donor partner with CYP2E1 was quite unexpected.

According to this analysis, the list of the most probable interaction partners of CYP2E1 (5–6 hits) includes P450s 2A6, 3A4, and 4F2, UGTs 1A6, 1A9, 2B4, 2B10, 2B15, and 2B17. The crosslinks of CYP2E1 with CYP4A11, CYP2C8, UGTs 1A1, 1A4, and 2B7, and cytochrome b_5_ were detected in four out of six experiments, which is also indicative of high-affinity interactions in these pairs. Other potential CYP2E1 interaction partners are fatty aldehyde dehydrogenase (FALDH), epoxide hydrolase 1 (EPHX1), disulfide oxidase 1α (Ero1α oxidase, ERO1L), and ribophorin II (RPN2).

## 4. Discussion

To our knowledge, this study is the first example of employing the CXMS technique with a membrane-incorporated protein activated by a photo-sensitive crosslinking reagent. The use of N-terminally truncated CYP2E1 containing C-terminal hexahistidine tag allowed for the isolation of the crosslinked aggregates and their further analysis with SDS-PAGE and untargeted proteomics. Along with that, the use of the truncated variant of CYP2E1 does not preclude its incorporation into the microsomal membrane. Furthermore, the similarity of the full-length and N-terminally truncated enzyme in the parameters of CYP2E1-dependent catalytic turnover and protein–protein interactions with other P450 enzymes demonstrated in our previous studies [35,37] suggest that the 3–20 truncations implemented in our construct does not change the mode of its interactions with the membrane considerably and do not affect the functional properties of the membrane-incorporated enzymes in any critical way.

This conclusion is in line with the current concepts on the mode of interactions of the microsomal P450 enzymes with the membrane, which are thought to be in large part determined by the regions between the N-terminal anchor and α-helix A [38,39,40], as well as by the hydrophobic surfaces in the regions of α-helices F’ and G’ [41,42,43], BC loop, and β1 sheet [44,45,46]. The most recent computational studies of P450 interactions with membranes performed in Rebecca Wade’s laboratory suggest that the latter two regions are essentially embedded into the lipid bilayer [44,46]. Furthermore, according to the X-ray structure of the full-length CYP51, the constrained orientation of the P450 catalytic domain relative to the membrane is dictated by a network of interactions of polar residues in the C-terminal part of the signal-anchor and the following proline-rich region [38], which are retained in our CYP2E1 construct, along with 11 out 23 residues of the membrane anchor sequence.

Application of our new CXMS strategy allowed us to demonstrate high-affinity interactions of alcohol-inducible CYP2E1 protein with P450s 2A6, 3A4, 4F2, and UDP-glucuronosyltransferases (UGTs) 1A and 2B. Our results also indicate CYP2E1 association with cytochrome b_5_, CYP2C8, and CYP4A11. In contrast to CYP2E1 interactions with P450s, UGTs, and cytochrome b_5_, which come as no surprise in the view of previous studies, the indications of CYP2E1 association with ERO1L, EPHX1, FALDH, and RPN2 were somewhat unexpected.

Similar to any crosslinking-based study of protein interactome, our approach may be prone to false positives caused by unspecific protein–protein contacts. The chance of capturing these transient contacts is especially significant in the crowded milieu of the microsomal membrane, where proteins interact via lateral diffusion. In our strategy for crosslink detection, the likelihood of false positives is diminished by relying on the reproducibility in multiple experiments with the use of two different crosslinkers (BPM and BPS). Nevertheless, the false positives cannot be ruled out, especially for highly abundant microsomal membranous proteins, such as ribophorin II. Therefore, further studies of potential interactions of CYP2E1 with ERO1L, EPHX1, FALDH, and RPN2 are needed to probe their specificity and possible metabolic role.

As noted above, the omission of NADPH-cytochrome P450 reductase (CPR) from the list of CYP2E1-crosslinked proteins was quite surprising. Analysis of the profiles of CPR distribution between SDS-PAGE zones detects the presence of CYP2E1-CPR crosslinks only in two out of six CXMS experiments (Table 3). This unsteady detection of CYP2E1-CPR was unexpected since both the BPM and BPS derivatives of the enzyme were shown catalytically active in 7-EFC deethylation and therefore retained their ability to interact with CPR. Notably, both instances of detecting crosslinks with CPR were observed with BPS-crosslinked samples, while crosslinking with BPM yielded no hits. Thus, a plausible explanation for the lack of a steady detection of crosslinks between CYP2E1-BPM and CPR may be the possible absence of cysteines available for modification with BPM near the CPR binding site in CYP2E1.

Another possible reason for the low frequency of detecting CYP2E1-CPR crosslinks is a severe shortage of CPR compared to the concentration of its electron acceptor partners [47]. Low abundance of CPR in the microsomal membrane results in stiff competition between multiple P450 species and other CPR-dependent enzymes, such as heme oxygenase [48,49], to form their complexes with this universal electron donor [50,51]. Furthermore, the formation of mixed oligomers between multiple P450 species is hypothesized to result in selective barring of some cytochrome P450 species from interactions with CPR due to specific organization of the P450 heterooligomers [8,9,10,11]. Following the logic of this hypothesis, the activating effect of CYP2E1 on CYP3A4 [26] and CYP1A2 [23] suggests that, when interacting with these highly abundant P450 species in the absence of its specific substrates, CYP2E1 predominantly occupies the so-called “latent” positions in heterooligomers, where its availability for interaction with CPR is limited [8,9]. As a result of these circumstances, the interactions of CYP2E1 with CPR may be disfavored.

Detection of multiple CYP2E1-crosslinked DMEs in our CXMS experiments corroborates the premise of a complex network of inter-protein interactions in the human drug-metabolizing ensemble. Identification of CYP3A4 as one of the most prominent interaction partners of CYP2E1 is in good agreement with our recent observation of a multifold activation of CYP3A4 in both CYP2E1-enriched microsomes and HLM preparations obtained from donors with a history of chronic alcohol exposure [26]. It also agrees with the results of our studies of CYP2E1-CYP3A4 interactions with LRET [35] and homo-FRET [26] techniques. Extensive interactions of CYP2E1 with CYP3A4 suggested by our results provide possible explanations for the alcohol-induced increase in the metabolism of diazepam and doxycycline [17,18,19], the substrates of CYP3A enzymes. Furthermore, CYP2E1 interactions with CYP2A6 suggested by our results may give a clue for the increased rate of metabolism of nicotine, a CYP2A6 substrate, in alcohol-dependent smokers [52].

Of particular interest is the observation of the crosslinking of CYP2E1 with CYP4F2 and CYP4A11, which are involved in the metabolism of arachidonic acid and its signaling metabolites. In particular, CYP4A11 plays a central role in the synthesis of vasoactive eicosanoids. Thus, its interactions with alcohol-inducible CYP2E1 may shed light on the mechanisms of alcohol-induced hypertension. Concurrently, the interactions of CYP2E1 with CYP4F2, the enzyme that initiates the inactivation of leukotriene B4 (LTB4), may impact cellular signaling by this pro-inflammatory eicosanoid. Thus, they may play a role in modulating inflammation by alcohol exposure [53]. In addition, potential interactions of CYP2E1 with FALDH, an enzyme catalyzing a subsequent step in LT4B degradation [54], may also be implicated in these effects.

The two P450 proteins most confidently identified as the protein–protein interaction partners of CYP2E1, namely CYP3A4 and CYP2A6, are among the most abundant P450 species in the ER of liver cells. The fractional content of CYP3A4 in HLM(LFJ) and HLM(LBA) is around 44%, while CYP2A6 contributes 16–21% to the total P450 pool [26]. CYP4F2 and CYP4A11 are also relatively abundant. Each constitutes 8–15% of the HLM P450 pool [55,56]. In contrast, the abundance of CYP2C8, another potential CYP2E1 partner identified in our study, is quite low. Its fractional content barely exceeds 1% in our HLM preparations [26]. This lesser abundance may cause less steady identification of its crosslinks with CYP2E1. It is also possible that our analysis missed the crosslinks of CYP2E1 with some other low-abundant P450 species.

Detection of the CYP2E1 crosslinks with UGTs 1A and 2B is in line with multiple reports on physical interactions between P450s and UGTs and their functional effects [57,58]. It should be noted that, in contrast to cytochromes P450 located at the cytoplasmic side of the ER, the catalytic domains of UGTs are exposed to the luminal side of the membrane. Therefore, direct interactions between the catalytic domains of UGTs and the heme domains of P450s do not seem possible. However, the transmembrane helix of UGT is known to pass through the membrane and expose a C-terminal portion of the protein of about 20 amino acids to the cytoplasmic side of the ER [59,60]. The studies on the interactions of CYP3A4 with UGT2B7 suggest that the contacting loci of the two proteins involve the cytosolic fragment of UGT [61,62] and the α-helix J of CYP3A4 [63]. Moreover, it was also suggested that the interactions might involve the contacts of membrane-incorporated part of UGT with the cytosolic domain of P450, which becomes possible through its more deep embedding into the membrane promoted by the P450–UGT association [64]. Therefore, the interactions between CYP2E1 with UGTs came as no surprise despite the apparent opposite orientations of these enzymes in the ER.

Most of the reports on P450-UGT interactions relate to the formation of UGTs 1A and 2B complexes with CYP3A4 [61,62,63,64,65,66], although the UGTs interactions with CYP1A1 [67] and CYP1A2 [68] were also detected. To our knowledge, the present study is the first that suggests the interactions between UGTs with CYP2E1. Validation of these interactions in a direct investigation and evaluation of their possible functional consequences may provide insight into the effects of alcohol exposure on the metabolism of drug substrates of UGTs, such as morphine and other opioids.

The proposed strategy of analyzing the effect of photo-activated crosslinking on protein distribution between the SDS-PAGE zones allowed us to point out the most probable protein interaction partners of the bait protein within the whole proteome of the microsomal membrane. The use of membrane incorporation of the tagged and chemically activated bait minimizes the risk of false positives characteristic of the strategies with nonspecific crosslinking. However, similar to any CXMS-based approach, our strategy cannot provide definitive proof of the physiological relevance of the detected interactions. It is also not suitable for obtaining information on the structure and stoichiometry of the protein–protein complexes. Answering these questions requires further investigations, which are now under development. The planned experiments involve using the CYP2E1 interaction partners as alternative baits in CXMS experiments with HLM and model microsomes containing recombinant full-length CYP2E1. Another series of studies will employ cleavable crosslinkers in the experiments with the pairs of purified proteins incorporated into reconstituted membranes to identify the interacting peptides and evaluate the structure of the protein complexes.

## 5. Conclusions

In total, our results demonstrate the high exploratory power of the proposed CXMS strategy and corroborate the concept of tight functional integration in the human drug-metabolizing ensemble through protein–protein interactions of the constituting enzymes. Further development of the proposed approach and its application for studying the interactome of other P450 enzymes will help elucidate the entire network of protein–protein interactions in the drug-metabolizing ensemble and understand the mechanisms of its integration into a multienzyme system.

## Figures and Tables

**Table 1:** Parameters of 7-EFC deethylation exhibited by intact and benzophenone-conjugated CYP2E1 incorporated into insect cell microsomes *.

Microsomal Preparation	*K_M_*, µM	*V*_max_, s^−1 a^
SS(2E1)	19.1 ± 5.2	0.099 ± 0.019
SS(CPR+b5)2E1	17.5 ± 3.7	0.122 ± 0.018
SS(CPR+b5)2E1-BPM	16.2 ± 0.7	0.104 ± 0.005
SS(CPR+b5)2E1-BPS	20.3 ± 3.3	1.51 ± 0.42

* The values given in the Table represent the averages of 2–4 individual experiments. The individual estimates of *K_M_* and *V*_max_ were obtained from fitting the substrate saturation profiles with the Michaelis–Menten equation. The values of *V*_max_ for CYP2E1-incorporated SS(CPR+b5) preparations are normalized per the concentration of CPR. In the case of SS(2E1), where CPR is present in excess, the turnover rate is calculated per the CYP2E1 concentration. The “±” values show the confidence interval calculated for *p* = 0.05.

**Table 2:** Preliminary identification of potentially crosslinked proteins *.

Protein Group	Gene Name	Number of Hits
Cytochromes P450	CYP2A6 and CYP2A7	4
	CYP2C8	5
	CYP2C9 and CYP2C19	5
	CYP3A4	4
	CYP4A11 and CYP4A22	4
UDP-glucuronosyltransferases	UGT1A1	4
	UGT1A4; UGT1A5	4
	UGT1A6	6
	UGT1A9, UGT1A8, UGT1A7, and UGT1A10	6
	UGT2B17	6
	UGT2B4	6
	UGT2B7	6
Other proteins of microsomal membrane	ALDH3A2	4
	EPHX1	4
	ERO1L	5
	FMO3	4
	RPN2	4

* The table shows the results of preliminary analysis of normalized peptide peak intensities observed for all microsomal membranous proteins with molecular masses of 45–85 kDa detected in MS/MS assays. The identification of potential crosslinks was based on the ratios of the normalized intensities observed in zones 3 and 4 (molecular masses of 85–155 kDa) to those detected in zones 1 and 2 (45–85 kDa). The occurrences (hits) where this ratio observed in the crosslinked sample was higher than that in the respective control were considered to indicate potential crosslinking. The table lists the proteins where these instances were encountered in at least four out of six individual CXMS experiments. The proteins listed in the same row could not be resolved in the MS/MS assays due to their high level of sequence similarity.

**Table 3:** Analysis of crosslinking-to-control ratios observed in six individual CXMS experiments *.

Protein	Crosslinking with BPM															Crosslinking with BPS															Total Number of Positives
	HLM(LFJ), Assay 1					HLM(LFJ), Assay 2					HLM(LBA)					HLM(LFJ), Assay1					HLM(LFJ), Assay 2					HLM(LBA)					
	55 ^a^	75	100	140	180	55	75	100	140	180	55	75	100	140	180	55	75	100	140	180	55	75	100	140	180	55	75	100	140	180	
CYP2A6 ^b^	0.3	0.7	∝	0.5	0	0.2	0.3	2	∝	0	0.1	∝	18	18	0	0.7	1.1	∝	0	0	0.5	0.6	1.2	∝	0	0.1	∝	∝	∝	∝	6
CYP2C8	0.7	1.2	∝	0.8	0.2	1.3	1.4	2.8	0.3	0	0.4	∝	6.6	0	0	1.4	1.2	∝	0	0	0.5	1.5	3.1	0.1	8.9	0.1	∝	4.7	3	8.3	4
CYP2C9 ^c^	1.2	0.5	∝	0.8	0.1	0.9	1.3	2.6	0.8	0	0.6	13	4.7	3.2	0	1.1	1.3	∝	0	0	0.4	2.9	4.4	1.6	∝	0.3	25	6.8	2	4.6	3
CYP3A4	0.7	2.1	∝	0.4	0	0.4	3.7	4	0.4	0	0.5	∝	∝	0	0	1	1.9	∝	0	0.7	0.2	4.2	3	0.7	∝	0	∝	∝	0	∝	5
CYP4A11 ^d^	0.7	1.2	∝	0.9	0	0.5	6.9	5	0	0	0.6	∝	0	0	0	1	0.6	∝	0	0	0.3	7.8	6	0.3	0	0.2	∝	2.4	0	0	4
CYP4F2	0.6	1	∝	∝	0	0.9	1.3	∝	0.2	∝	1.2	13	0	0	0	1	0.3	∝	0	0	0.3	5	∝	1.2	∝	1.2	∝	0	0	0	5
CYB5A	∝	0.9	0	0	0	∝	0.9	∝	0	0	0	0	0	0	0	∝	0.8	0	0	0	∝	0.6	∝	∝	∝	0	0	0	0	0	4
CPR	3.9	1.2	0.2	3.3	0	0.7	∝	0.1	0.1	0	1.7	11	0	0	0	11	1.1	0.3	0	0	3.5	∝	0.1	0.1	∝	19	0.8	129	0.0	∝	2
UGT1A1	1	0.7	∝	0.5	0	1.5	0.3	6.4	0.4	0	1	0	0	0	0	0.5	2.5	∝	0	0	1	0.6	6.3	0.4	∝	0	∝	0	0	0	4
UGT 1A4 ^e^	0.8	0.6	∝	0.6	∝	1	0.3	3.9	1.2	0	1	0	0	0	0	0.5	1.4	∝	0	∝	0.6	0.9	5.2	1.6	∝	0.5	∝	0	0	0	4
UGT 1A6	0.6	1.7	∝	1.3	∝	0.7	0.9	3	1.3	∝	0.7	∝	0	∝	0	0.7	1.4	∝	0	0	0.5	0.6	2.7	2.8	∝	0.6	∝	∝	∝	∝	6
UGT 1A9 ^f^	0.6	∝	∝	∝	0	0.7	∝	∝	∝	0	0.5	∝	∝	1	0	0.6	∝	∝	0	0	0.3	∝	∝	∝	0	0.3	∝	∝	1	∝	6
UGT 2B10	0.2	3.1	∝	0	0	0.2	∝	2	∝	0	0.6	∝	0	0	0	1.1	0	0	0	0	0	0	3	0	∝	0	∝	0	0	0	5
UGT 2B15	0.6	0.9	∝	2.1	∝	0.7	∝	2.9	1.1	0	0.6	∝	0	0	0	1	1.5	∝	0	0	0.2	∝	5.3	1.4	∝	0	0	0	0	0	5
UGT 2B17	0.8	1	∝	0.6	1.5	0.6	0.4	7	0.7	14	0.5	∝	8.3	∝	0	1	1.5	∝	0	0	0.4	0.4	3.4	1.6	36	0	∝	6.8	∝	∝	5
UGT 2B4	0.7	0.9	∝	0.9	2	0.5	5	4.4	1.9	∝	0.7	∝	1.7	∝	0	1	0.9	∝	0	0	0.2	12	3	4.9	∝	0.4	∝	0	∝	0	6
UGT 2B7	0.6	0.9	∝	1	1.2	0.5	1.2	2.3	0.8	3.1	0.7	∝	∝	7.6	0	0.6	1	∝	0	4	0.2	3.3	2.3	1.4	3.3	0.1	∝	∝	8.4	∝	4
ALDH3A2	0.4	0.6	∝	∝	0	0.3	∝	5	3.4	0	0	0	0	0	0	0.8	0.8	∝	0	0	0.2	∝	6.5	2.3	∝	0	0	0	0	0	4
EPHX1	0.2	0.6	∝	0.6	1.5	0.7	∝	1	0.4	0.8	0.2	∝	1.6	0.8	∝	0.2	0.4	∝	0.4	2.7	0.9	∝	0.8	1.2	0.7	0.2	∝	0.7	0.9	∝	6
ERO1L	0.8	1	0	∝	0	0.7	∝	0	∝	0	0	0	0	0	0	0.7	1.8	∝	0	0	0.4	∝	∝	∝	∝	0	0	0	0	0	4
RPN2	0.7	1.3	∝	∝	∝	0.4	∝	∝	∝	∝	0.8	∝	∝	∝	∝	0.9	1.8	∝	∝	∝	0.7	∝	∝	∝	∝	0.4	∝	∝	∝	∝	6
CES1	1.4	1.6	0.4	1.1	1.4	1.2	0.8	0.3	0.9	3.3	3.5	1.2	0.7	0.6	0.1	1.1	1.5	0.4	1.4	1.9	1.2	0.5	0.5	1.2	3	2.1	2.7	1.1	0.4	1.7	0
P4HB	1.1	1.5	0.2	5.7	6.4	1.1	1	0.3	3.8	1.9	0.8	11	6.3	0.8	2.6	0.8	1.6	0.3	6.4	13	0.9	0.5	0.6	5.5	4.6	0.5	28	9.7	0.8	15	0

* The table shows the ratios of peak intensities in the SDS-PAGE zones of crosslinked samples to those in control with unlabeled CYP2E1. The infinity sign (∞) indicates the cases where no protein was found in control while present in the crosslinked sample. The ratios >30 observed in the zones with molecular masses higher than that of the protein itself are shown in bold as indicative of crosslinking. Identifiers of proteins with >3 hits are shown in bold. ^a^ This row shows the approximate average molecular masses corresponding to the SDS-PAGE zones. The masses are expressed in kilodaltons (kDa). ^b^ This row corresponds to both CYP2A6 and CYP2A7, which could not be reliably resolved in LC-MS/MS assays. ^c^ This row corresponds to both CYPC9 and CYP2C19. ^d^ This row corresponds to both CYP4A11 and CYP4A22. ^e^ This row corresponds to both UGT 1A4 and UGT 1A5. ^f^ This row corresponds to the total of UGT 1A7, 1A8, 1A9, and 1A10.

## Data Availability

Raw results of MaxQuant analysis of LC-MS/MS data are available in Table S1 found in the Supplementary material to this publication. Other research materials may be obtained from the authors upon a reasonable request.

## Supplementary Materials

The following is available online at https://www.mdpi.com/article/10.3390/biom12020185/s1: Table S1, Results of the individual CXMS experiments.
